# Ceruminal diffusion activities and ceruminolytic characteristics of otic preparations – an *in-vitro* study

**DOI:** 10.1186/1746-6148-9-70

**Published:** 2013-04-10

**Authors:** Jessica Stahl, Stefanie Mielke, Wolf-Rüdiger Pankow, Manfred Kietzmann

**Affiliations:** 1 Department of Pharmacology, Toxicology and Pharmacy, University of Veterinary Medicine Hannover, Foundation, Buenteweg 17, 30559, Hannover, Germany; 2 Vétoquinol GmbH, Parkstraße 10, 88212, Ravensburg, Germany

**Keywords:** Canine otitis externa, Cerumen, Lipids, Otic preparations, Ear cleaner, Diffusion activity, Ceruminolysis

## Abstract

**Background:**

An *in-vitro* setup was established in order to determine a) the diffusion activities of eight otic preparations (Aurizon®, Eas Otic®, Epi Otic®, Otifree®, Otomax®, Panolog®, Posatex®, Surolan®) through synthetic cerumen, and b) the ceruminolytic capacity and impregnation effects of these products. The main lipid classes of canine cerumen produced with moderate, non-purulent otitis externa were determined by thin layer chromatography and were subsequently used to produce a standardised synthetic cerumen (SCC). SCC was filled into capillary tubes, all of which were loaded with six commercially available multipurpose otic medications and two ear cleaners, each mixed with two markers in two experimental setups. These two marker compounds (Oil red O and marbofloxacin) were chosen, since they exhibit different physicochemical drug characteristics by which it is possible to determine and verify the diffusion activity of different types of liquids (i.e. the otic preparations). A synthetic cerumen described in the literature (JSL) was also used for comparison as its lipid composition was different to SCC. The diffusion activities of the otic preparations through both types of synthetic cerumen were studied over 24 hours. A second *in-vitro* experiment determined both the ceruminolytic activity and impregnation effect of the otic preparations by comparing the weight loss or weight gain after repeated incubation of JSL.

**Results:**

Canine cerumen is mainly composed of triglycerides, sterol esters, fatty acid esters and squalene. The diffusion experiments showed a high diffusion efficacy along with a high impregnation effect for one test product. All the other products exhibited a lower diffusion activity with a mild to moderate impregnation effect. A mild ceruminolytic activity was observed for the two ear cleaners but not for any of the otic medications.

**Conclusions:**

The present study demonstrates that there are significant differences in the diffusion characteristics and ceruminolytic properties of the eight tested otic preparations.

## Background

Canine otitis externa (OE) is one of the most common diseases in small animal practice [1,2]. It is a syndrome with a multifactorial aetiology. The factors are classified as predisposing (increase the risk of otitis), primary (directly induce otitis), secondary (contribute to otitis only in an abnormal ear or in conjunction with predisposing factors), and perpetuating (result from inflammation and pathology in the ear, prevent resolution of otitis) [3-6].

In the external ear canal, cerumen influences the effective barrier function for the underlying cutaneous epithelium. The presence of cerumen is normal and is mandatory for a physiological movement of microorganisms or foreign substances from the deep ear canal towards its external opening [7,8]. Cerumen is a complex mixture of desquamated keratinocytes and debris in combination with secretions of both the ceruminous and the sebaceous glands of the external ear canal [9,10]. Although gland density and distribution differs markedly between canine individuals, a general pattern has been observed with sebaceous tissue increasing gradually from the proximal to the distal parts of the canine ear canal, whilst the number of ceruminous glands decrease [11].

In chronic OE, the quantity of ceruminous glands increases significantly, caused by the inflammatory process [12] and gland hyperplasia can be observed. Another study [11] found no relevant differences between inflamed and healthy ears with regard to the gland distribution and hair follicle density despite the fact that the glands and hair follicles became hyperplastic in affected ears. However, the lipid content of cerumen from inflamed ears is significantly lower than from healthy ears [7]. Regarding the lipid composition of human cerumen, several studies have demonstrated high amounts of cholesterol, sterol ester and fatty acids [9,10,13], but there is a lack of information about canine cerumen lipids except for fatty acids which are found regularly and in high quantities in OE-affected ears [14].

Topical therapy is an important part of the treatment of inflamed ears. Multipurpose products (otic preparations) exhibiting antibacterial, antifungal or anti-inflammatory properties are frequently indicated, particularly initially, because of the mix of microorganisms, inflammation and sometimes, parasites that are present at the time of diagnosis [1,3]. According to the package leaflets of such otic preparations, the topical treatment should be performed after mechanical (not supported by many specialists) or chemical removal of cerumen from the external ear canal [15,16]. However, cerumen may remain in the external ear canal and can reduce treatment effectivity. Otic preparations with active ingredients against OE should remain inside the external ear canal for an adequate amount of time in order to provide adequate antibacterial, antifungal and anti-inflammatory effects. Furthermore, ceruminolytic activity and diffusion through cerumen residues are beneficial for high efficacy on the skin surface.

The objective of the present study was to determine the diffusion activity of eight commercial otic preparations through cerumen. Therefore, an *in-vitro* method was developed using synthetic canine cerumen where diffusion activities could be measured by applying two marker compounds with different physicochemical characteristics. In addition, the impregnation effect and ceruminolytic activity of the test products were studied in the *in-vitro* setup.

## Results

The lipid analysis of canine cerumen revealed high levels of squalene, fatty acid esters, sterol esters and triglycerides. All other lipid classes were found with amounts smaller than 2.5% of the total cerumen lipids (Table 1).

**Table 1:** Lipid composition of canine cerumen of 12 healthy dogs and 12 dogs with acute moderate, non-purulent OE

**Lipid**	**% of Total lipids**			
	**Healthy dogs**		**OE dogs**	
	**Mean**	**Standard deviation**	**Mean**	**Standard deviation**
Alkanes	n.q.	n.q.	n.q.	n.q.
Cholesterol	1.68	0.66	1.55	1.28
Cholesteryl sulphate	0.12	0.13	0.08	0.13
Ceramide [AP]	0.78	0.38	0.71	0.75
Ceramide [AP/AS]	0.48	0.23	0.35	0.35
Ceramide [AS]	0.31	0.22	0.25	0.27
Ceramide [EOS]	n.d.	n.d.	n.d.	n.d.
Ceramide [NP]	0.45	0.22	0.48	0.34
Ceramide [NS]	0.33	0.27	0.66	0.56
Fatty acid ester	8.50	3.36	5.79	4.47
Free fatty acids	1.50	0.62	1.42	1.21
Galactocerebrosides	0.31	0.34	0.23	0.50
Phospholipids	0.08	0.12	0.29	0.60
Squalene	2.08	2.41	3.19	2.58
Sterol ester*	14.30	10.24	6.97	5.07
Triglycerides	21.01	6.08	18.89	13.99

The quantities are expressed as means and standard deviations after determination with thin layer chromatography, n.d. = not detectable, n.q. = not quantifiable; asterisks indicate p < 0.05 (*t*-test with Mann Whitney test, two-tailed).

**Figure 1:**
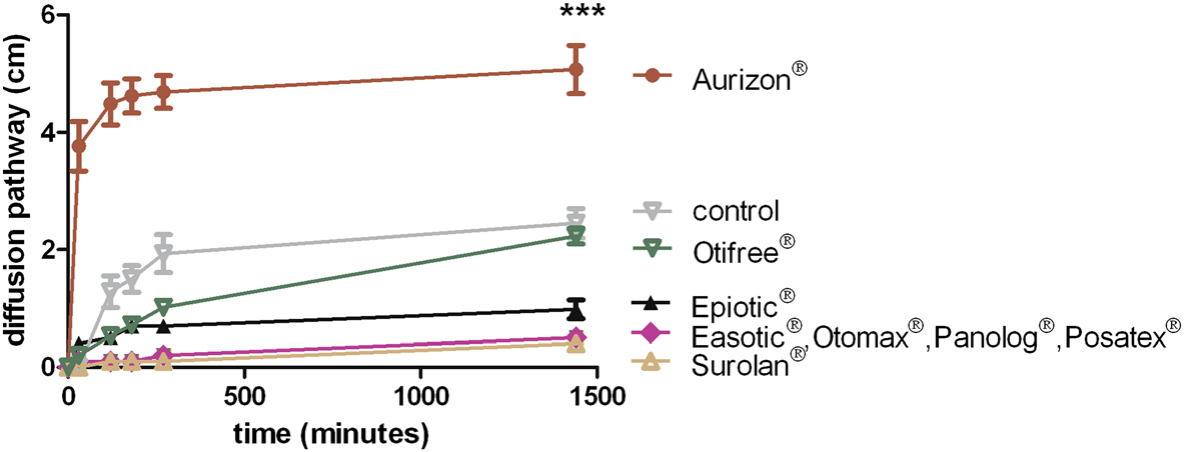
Diffusion of Oilred O supplemented to eight otic preparations through synthetic canine cerumen (SCC), means ± standard deviations, n = 6, asterisks (***) indicate p < 0.001 after 1440 h (Aurizon® vs. seven other formulations and control).

**Figure 2:**
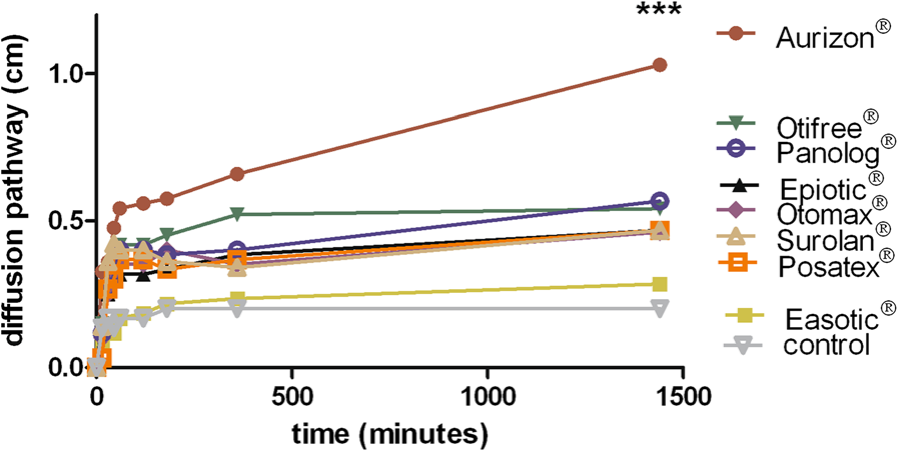
Mean diffusion of Oilred O supplemented to eight otic preparations through synthetic canine cerumen (JSL), n = 6, asterisks (***) indicate p < 0.001 after 1440 h (Aurizon® vs. seven other preparations and control).

The extent and velocity of the diffusion activities in SCC are shown in Figure 1. Oil red O added to the ear cleaners Epi Otic® and Otifree® exhibited a slower diffusion activity (1.0 and 2.2 cm) compared to Aurizon® (5.1 cm) and the control (2.5 cm). Surolan® showed the lowest diffusion (0.4 cm) of all the investigated otic preparations (Figure 1). The diffusion through JSL showed comparable results except for the control (Figure 2). Aurizon® diffused to the greatest degree (1.05 cm after 24 hours), followed by Otifree® (0.54 cm). The control diffused only 0.2 cm.

**Figure 3:**
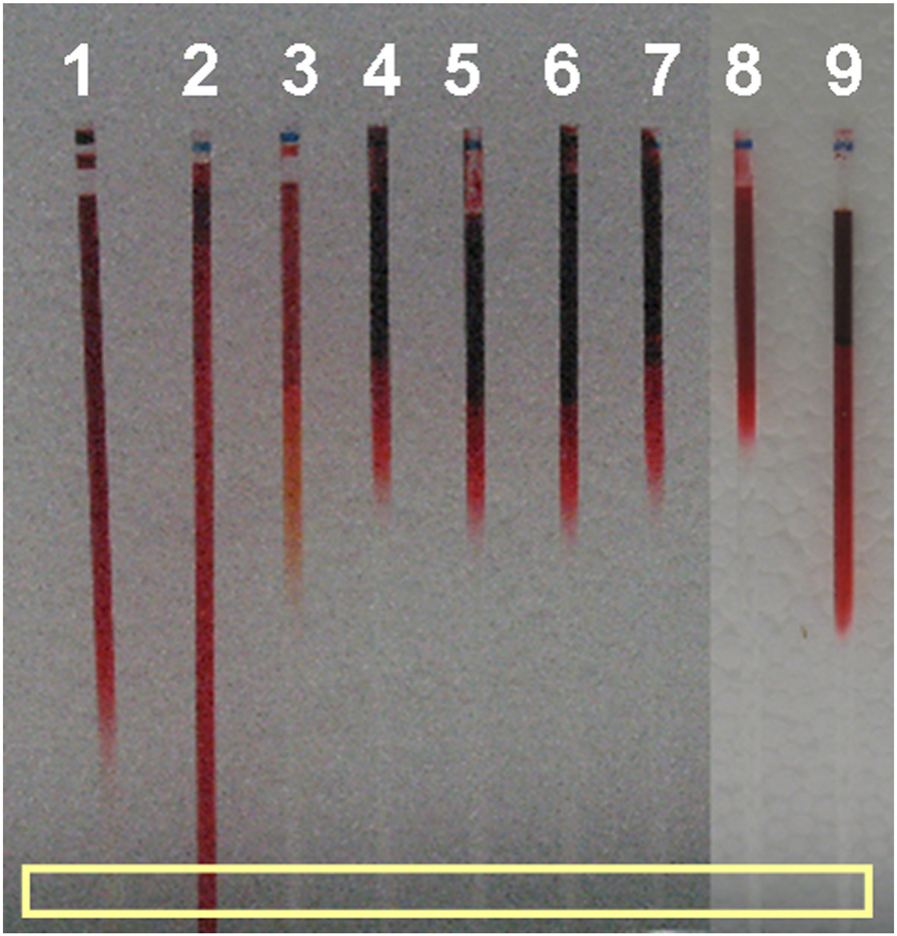
**Oil red O diffusion through synthetic canine cerumen (SCC) after 24 hours, 1 = control (SCC), 2 = Aurizon®, 3 = Epi Otic®, 4 = Eas Otic®, 5 = Panolog®, 6 = Posatex®, 7 = Otomax®, 8 = Surolan®, 9 = Otifree®.** The yellow box indicates the approximate area of SCC sampling for the HPLC analysis of supplemented marbofloxacin.

The determination of marbofloxacin in the SCC revealed a positive correlation of marbofloxacin with the Oil red O colour in case of Aurizon® (5 cm diffusion lengths). No marbofloxacin was determined at this position in any of the capillary tubes filled with the other seven otic preparations (Figure 3).

**Figure 4:**
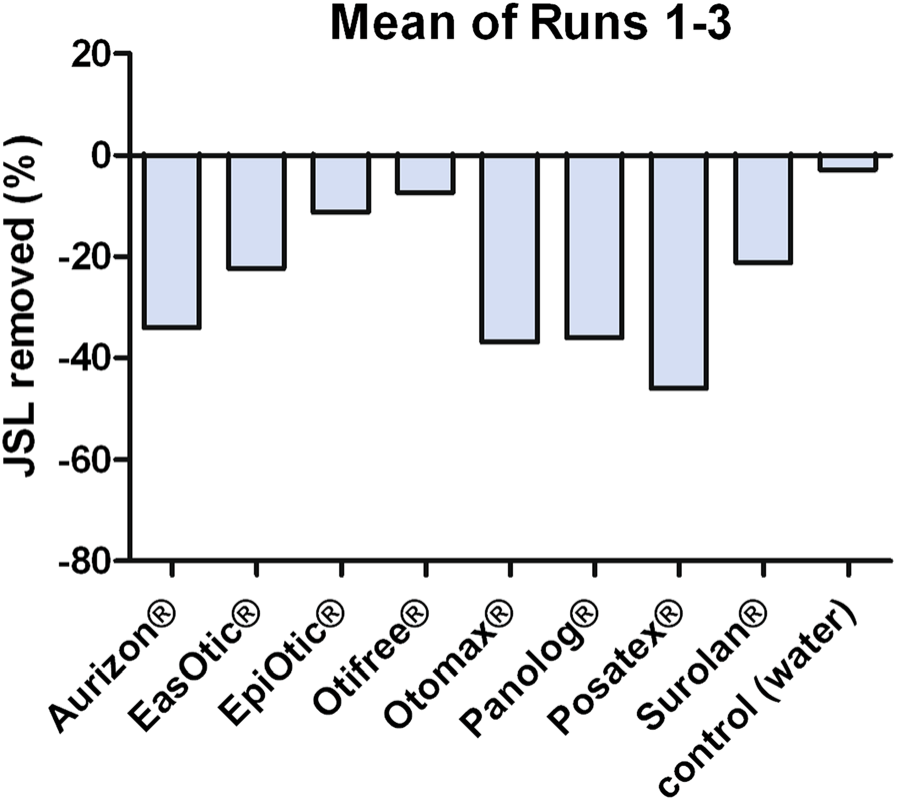
Mean percentage of standardised synthetic cerumen (JSL) removed after Tests 1–3, n = 4 loadings; positive values indicate ceruminolysis, negative values indicate that there has been impregnation of the product.

**Figure 5:**
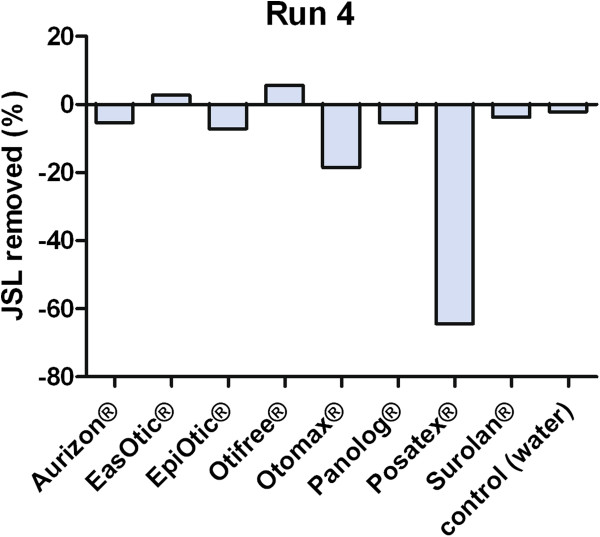
**Mean percentage of standardised synthetic cerumen (JSL) removed after Test 4; weighing was performed after washing with cold water in order to remove all product residues, n = 4 loadings per Test.** Positive values indicate ceruminolysis; negative values indicate that there has been impregnation of the product.

The examination of the ceruminolytic activity of the test products showed that Eas Otic® and Otifree® were able to remove some cerumen after incubation, though with a high variability in the Eas Otic® samples (Table 2, Figures 4 and 5). All products except the ear-cleaning solutions (Otifree®, Epi Otic®) and the control exhibited a weight gain indicating an impregnation by either incorporation or adhesion.

**Table 2:** Percentage of standardised synthetic cerumen (JSL) removed after each run (Test)

**Test product**	**Weight removed (%)**				
	**Test 1**	**Test 2**	**Test 3**	**Mean of tests 1-3**	Test 4*
	**Mean ± SD**	**Mean ± SD**	**Mean ± SD**		**Mean ± SD**
Aurizon®	−42.7 ± 14.4	−29.8 ± 4.2	−29.3 ± 4.9	−34	−5.3 ± 3.8
Eas Otic®	−46.2 ± 2.9	−3.0 ± 8.7	−17.7 ± 14.7	−22.3	2.7 ± 6.5
Epi Otic®	−9.2 ± 3.1	−12.4 ± 3.2	−11.8 ± 2.5	−11.2	−7.1 ± 2.0
Otifree®	−8.1 ± 4.3	−3.5 ± 2.7	−10.3 ± 5.6	−7.3	5.6 ± 1.2
Otomax®	−68.6 ± 6.7	−19.3 ± 10.0	−22.7 ± 6.3	−36.9	−18.5 ± 3.0
Panolog®	−57.8 ± 10.4	−15.5 ± 5.8	−34.7 ± 8.4	−36	−5.4 ± 7.1
Posatex®	−58.2 ± 10.8	−32.4 ± 11.8	−47.4 ± 9.9	−46	−64.4 ± 5.5
Surolan®	−20.4 ± 8.6	−19.4 ± 7.5	−23.5 ± 8.8	−21.1	−3.7 ± 7.1
Control (water)	−2.5 ± 1.1	−3.4 ± 1.6	−2.6 ± 1.0	−2.8	−2.1 ± 3.3

* Weighing was performed after washing with cold water (mean ± standard deviation), n = 4 loadings per test.

Positive values indicate ceruminolysis; negative values indicate that there has been impregnation of the product.

## Discussion

Due to pathological changes in the ceruminous glands, the total amount of lipids (% by weight) of canine cerumen of OE ears has been described to differ significantly from healthy ears [7]. In our analysis, however, the detailed analysis of various lipid classes in canine cerumen from healthy and inflamed ears demonstrated comparable lipid compositions except for sterol ester (Table 1). The lipid composition of the SCC used in the present study differed from that implemented by Sánchez-Leal et al. [17]. However, these investigators used synthetic cerumen based on the average results of human and canine cerumen studies [9,10,14] with diminished concentrations of oleic acid to obtain adequate consistency and malleability. Thus, the main components in their synthetic cerumen were fatty acids (myristic acid, palmitic acid and oleic acid). In contrast, triglycerides dominated in our cerumen composition derived from the cerumen of OE-affected dogs. This difference may be due to variant methodologies of extraction and analysis or the time of the year when the sampling was done. Furthermore, it is known that storage before analysis can promote lipase activity in cerumen with the result that the triglycerides are metabolised to fatty acids. In our study, the lipids were immediately extracted, but information about storage times is not available in the literature data on which the synthetic cerumen (JSL) described by Sánchez-Leal et al. [17] was based. To overcome this lack of information, the diffusion studies were performed using both these lipid mixtures. The results were found to be similar: in the case of persistent cerumen films in the auditory canal, Aurizon® would exhibit the fastest diffusion through the cerumen lipids.

The lipid classes present in the synthetic cerumen resulted in a particular lipid architecture [18], which had to be softened and dissolved to provide adequate space for diffusion in our experiments. It seems that the content of sorbitan oleate in Aurizon® with a molecular structure similar to several skin penetration enhancers like sucrose oleate [19] plays a major role in this respect. Nevertheless, it cannot be ruled out that the other excipients used in Aurizon® such as the medium-chain triglycerides, silicon dioxide or propyl gallate interact with the cerumen lipids and promote diffusion activity. Since all the other otic preparations tested, except for the ear cleaners, contain liquid paraffin, the lower diffusion activity of these products may even be due to its supplementation. Liquid paraffin is a mixture of various alkanes, which are only marginally detectable in canine cerumen, which may be a reason why liquid paraffin-containing products exhibit low diffusion activities. In contrast, the composition of Aurizon® is more comparable to the composition of canine cerumen with regard to its triglyceride content and, therefore, it is not surprising that Aurizon® showed a better diffusion activity.

Penetration through lipid-rich domains in the skin is mainly determined by the lipophilicity of a drug. In addition, the melting point and the molecular weight can influence the diffusion process [20]. Oil red O was chosen in order to visualise the diffusion of a lipophilic drug (LogP 7.6) with a moderate molecular weight of 409 g/mol and a melting point of 120°C. For comparison, the diffusion of a hydrophilic drug (marbofloxacin) with similar molecular weight (362 g/mol) and a melting point of 268°C was selected. Marbofloxacin has comparable diffusion behaviour to Oil red O, although a main physicochemical characteristic for diffusion through lipid layers, its lipophilicity, differs markedly. The HPLC analysis ruled out that only lipophilic compounds like Oil red O diffused through cerumen. From this result it can be assumed that active ingredients with physicochemical drug characteristics similar to the marker compounds used in the diffusion test would exhibit similar diffusion behaviour.

The ceruminolysis results showed that the high viscosity of some of the products led to a weight gain due to impregnation (Tests 1–3). Since impregnation can be caused either by incorporation of the product and disintegration of cerumen lipids or by adhesion to the cerumen surface, the products were removed from the tubes in Test 4 thereby allowing the evaluation of the ceruminolytic effects of the viscous products. Aurizon®, Otomax®, Panolog® and Posatex® exhibited a high potency to impregnate the cerumen, while Eas Otic® and Surolan® showed an intermediate potency. The ear cleaners Epi Otic® and Otifree® had very little impregnation potency, as expected. These differences may be due to the different amounts of viscous excipients like liquid paraffin (Eas Otic®, Otomax®, Posatex®, Surolan®, Panolog®) or medium-chain triglycerides (Aurizon®), all of which are not included in the ear cleaners.

Since the investigated otic preparations (with the exception of Epi Otic® and Otifree®) have to remain in the external ear canal to exhibit optimal antimicrobial, antifungal or antiparasitic activity, a high impregnation potency will contribute to a good efficacy. With regard to the results of the diffusion study, Aurizon® seems to provide ideal conditions for its active compound marbofloxacin to get into the cerumen. On the other hand, none of the products (except for Eas Otic® with a high standard deviation) performing a high or intermediate impregnation effect exhibited any ceruminolytic activity. A slight weight loss was only found with Otifree® and Eas Otic®. It can just be assumed that propylene glycol (excipient in Otifree®) caused disturbances in the lipid architecture of the cerumen and elimination, since propylene glycol has been described as being able to modify skin penetration of topically applied drugs such as tenoxicam or bupranolol [21-24].

### Limitations

The penetration of the active components of the tested products is based on the assumption that they have similar physicochemical properties as Oil red O or marbofloxacin. Both these compounds have comparable molecular weights (362–409 g/mol) and melting points (120-268 C). The penetration effect of the products was evaluated assuming that penetration is mainly influenced by the lipophilicity of the drug (similar to skin permeability through lipid-rich domains). Oil red O and marbofloxacin are extremely different concerning their lipophilicity. They were chosen to cover a wide range of different lipophilicities. Nevertheless, the eight otic preparations also contain active components with other physicochemical qualities, which may not be covered by the present study. Furthermore, missing components of natural cerumen like proteins or keratinocytes may affect the penetration of active components as well, due to their breaking up the lipid matrix and unknown interactions with the active components.

The ceruminolytic activity was evaluated on the basis of the lipolytic effect of the products. The addition of other components of natural cerumen like keratinocytes or proteins would provide results closer to the *in-vivo* situation, albeit possibly with increased test variability. Due to the lack of information on these components of natural cerumen, further studies are required.

## Conclusions

In conclusion, the present study is the first investigation providing detailed information about the lipid composition of canine cerumen from healthy and inflamed ears. The results of the *in-vitro* assays demonstrate a high variability of otic preparations with respect to their diffusion potency through canine cerumen and their ceruminolytic effect. Of the six multipurpose otic products tested, only Aurizon® exhibited a fast diffusion through synthetic cerumen and an impregnation effect, though without any ceruminolytic activity. A slight ceruminolytic effect was observed for one test product, the aqueous ear cleaner Otifree®; less consistent results were found for the ear cleaner Epi Otic®.

## Methods

### Lipid analysis of canine cerumen

Canine cerumen was collected with lipid-free cotton buds from dogs (n = 12) of different sexes, breeds and ages, all of which had an acute, moderate OE. Purulent OE ears were excluded from the study (gross examination without cytology). Information about the aetiologies was not available. The dogs were owned by the Clinic for Small Animals, University of Veterinary Medicine Hannover, Foundation, and had not been pretreated with any otic preparations. Cerumen of clinically healthy dogs (n = 12) of different sexes, breeds and ages, was collected as well.

**Figure 6:**
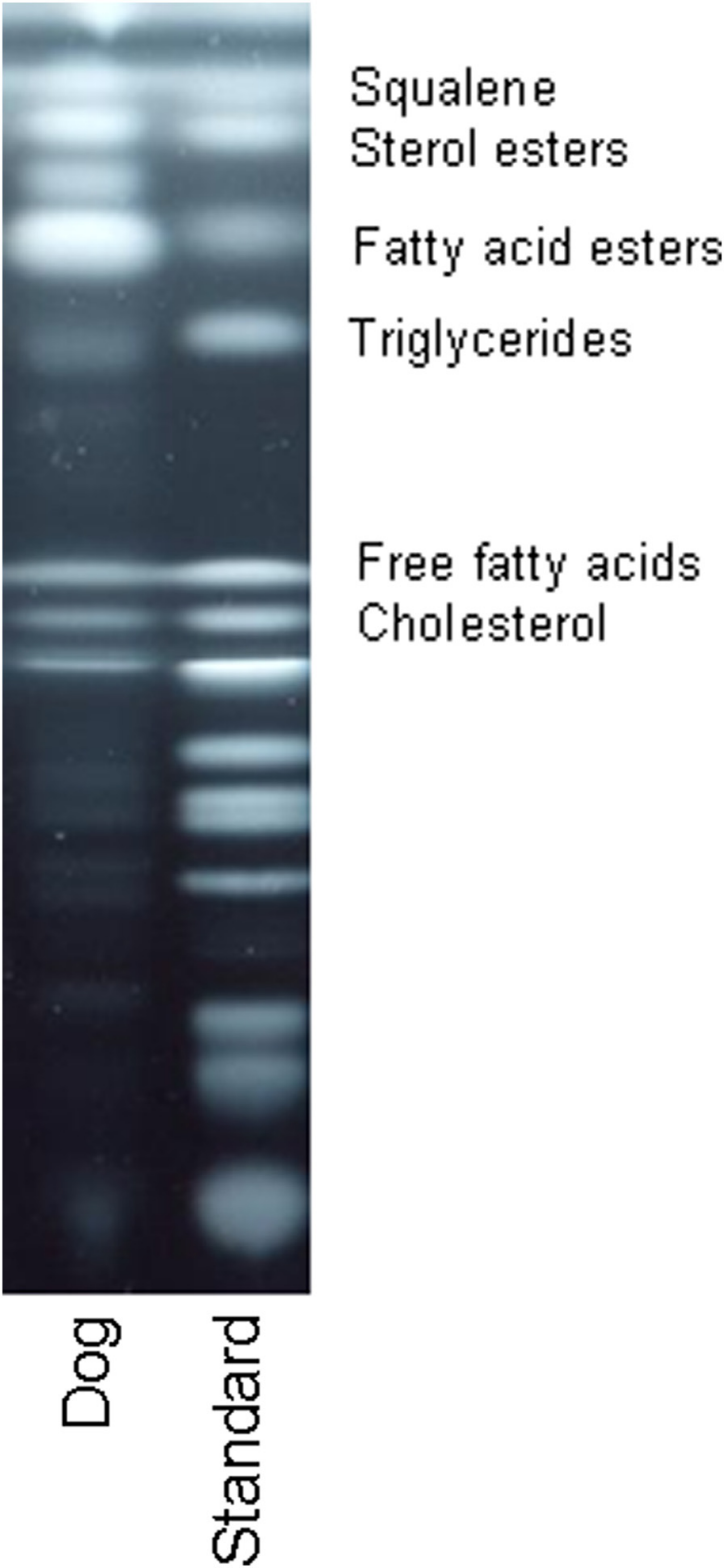
Thin layer chromatography presenting the composition of canine cerumen in comparison to lipid standards.

Lipid extraction was performed according to Reiter et al. [25]. In brief, the ceruminal lipids of each dog were extracted from the cotton bud with hexane/ethanol (95/5; V/V) for 20 minutes by sonication. The supernatant was collected and the procedure was repeated one more time. After evaporation of the solvent, the total amount of lipid was determined. The analysis of the different lipid classes of each dog was performed by thin layer chromatography (TLC, Figure 6). The extracted lipids were dissolved in chloroform/methanol (2:1, v/v) and were applied to TLC plates (Merck, Darmstadt, Germany) using a previously described methodology [26]. Co-migrated lipid standards were used to identify and quantify the different lipid classes.

### Synthetic canine cerumen (SCC)

SCC was used in order to provide large amounts of cerumen for the *in-vitro* studies without the effects of other natural cerumen ingredients like keratinocytes and proteins. According to authors’ knowledge, the composition of canine cerumen components has not been fully described as yet. Thus, high variability was avoided by using SCC based on the essential lipid classes of canine cerumen, i.e. components with >2.5% of the total lipids determined by TLC analysis were used: 9.16% squalene, 16.62% fatty acid ester, 19.98% sterol ester and 54.24% triglycerides (all from Sigma-Aldrich, Steinheim, Germany).

### Diffusion activity

SCC (47–57 mm^3^) was filled into glass capillaries (diameter 1.15 ± 0.05 mm, length 75 ± 1 mm). The otic preparations (Table 3) were added (16–21 mm^3^) onto the SCC in the capillary tubes. Before starting the experiments, each of the products was mixed with two markers (two independent experiments with either Oil red O or marbofloxacin supplementation, Table 4), which exhibit different physicochemical drug characteristics that enable the extent and velocity of product diffusion through the SCC to be determined.

**Table 3:** Otic preparations with their active ingredients, excipients and galenic formulation; the asterisks indicate the ear cleaners

**Product**	**Registered composition**		**Galenic formulation**	**Marketing authorisation holder**
	**Active ingredients**	**Excipients**		
Aurizon^®^	Marbofloxacin, clotrimazole, dexamethasone acetate	Propyl gallate (E310), sorbitan oleate, silicon dioxide, medium-chain triglycerides	suspension	Vétoquinol GmbH, Ravensburg, Germany, Batch number 1A18750/03605877138794
Eas Otic^®^	Hydrocortisone aceponate, miconazol nitrate, gentamicin sulphate	Liquid paraffin	suspension	Virbac Tierarzneimittel GmbH, Bad Oldesloe, Germany, Batch number 3HLS
Otomax^®^	Gentamicin sulphate, betamethasone valerate, clotrimazole	Liquid paraffin, hydrocarbon gel	suspension	Intervet Deutschland GmbH, Unterschleissheim, Germany, Batch number APNA 1231
Panolog^®^	Triamcinolone acetonide, neomycin sulphate, nystatine, thiostreptone	Plastibase 10W (95% liquid paraffin, 5% polyethylene)	suspension	Novartis Tiergesundheit GmbH, Munich, Germany, Batch number 0404811922901734721
Posatex^®^	Orbifloxacin, momethasone furoate, posaconazole	Liquid paraffin, hydrocarbon gel (5% polyethylene in 95% mineral oil), lauric acid	suspension	Intervet Deutschland GmbH, Unterschleissheim, Germany, Batch number 1117101
Surolan^®^	Prednisolone acetate, polymyxin B sulphate, miconazol nitrate	Silicon dioxide, liquid paraffin	suspension	Janssen Animal Health, Neuss, Germany, Batch number BHB1L02
Epi Otic^®^*		Salicylic acid, dictyle sodium, sulfosuccinate, chloroxylenol, EDTA, monosaccharides	solution	Virbac Tierarzneimittel GmbH, Bad Oldesloe, Germany, Batch number 3EP8
Otifree^®^*		Propylene glycol, cremophor, calendula extract, basil oil, water	solution	Vétoquinol GmbH, Ravensburg, Germany, Batch number 0B4953K

**Table 4:** Physicochemical properties of Oil red O and marbofloxacin

	**Oil red O**	**Marbofloxacin**
Molecular weight (g/mol)	409	362
LogP	7.577	−0.835
Melting point (°C)	120	268

Marker 1: Oil red O (10 mg/ml) was used to visualise the diffusion distance of each of the otic preparations by measuring the diffusion pathway of Oil red O optically after 24 hours with a ruler. Oil red O in SCC served as the control. The capillaries were stored at 32°C in a water bath to imitate the physiological conditions of the external ear canal of the dog. An identical experiment was performed with synthetic cerumen (JSL) as used by Sánchez-Leal et al. [17], which had the composition: 33.6% myristic acid, 33.6% palmitic acid, 9.4% oleic acid, 10.9% cholesterol and 12.5% squalene. Each otic preparation was studied twice, i.e. in six glass capillaries on SCC (n = 6) and on JSL (n = 6).

Marker 2: Marbofloxacin (10 mg/ml) was chosen as the second marker, which is also an active compound of one test product (Aurizon®). A validated high performance liquid chromatography method (HPLC) [27] was used to detect the diffusion of the active ingredient (marbofloxacin) through the SCC in comparison to the dye Oil red O. For this purpose, marbofloxacin was added to each test product before it was applied to the SCC. After incubating for 24 hours, samples were taken from the 5 cm diffusion distance for each test product (Figure 3).

Extraction was performed by adding 300 μl NaOH (5 N) and shaking for 10 minutes. After centrifugation at 230 g for 10 minutes, 200 μl supernatant was taken and was mixed with 200 μl HCl (5 N) before analysis. A Beckman HPLC instrument (Munich, Germany) with the following parts was used: a 126 pump with a flow rate of 1.0 ml/min, a 507 autosampler with 100 μl injection volume, a RF535 detector (Shimadzu, Kyoto, Japan) with an excitation wavelength of 280 nm and a detection wavelength of 450 nm, a CC 250/4 NUCLEODUR 100–5 C18ec column and a column heater (40 C; Spark Holland, Emmen, The Netherlands).

### Ceruminolytic activity and impregnation effect

The ceruminolytic activity of each otic preparation was studied by using JSL as outlined by Sánchez-Leal et al. [17]. This was chosen because the texture of the SCC based on the main lipid classes of canine cerumen was too liquid. 250 mg JSL was filled in plastic tubes at 45°C (the solid cerumen became liquid) and was cooled down to 32°C.

The initial weight of the tubes with JSL was determined. Subsequently, 1 ml of the test product was added. After 20 minutes incubation on a shaker (150 rpm, 32°C) to simulate contact time and the head shaking occurring in a real canine ear, the tubes were inverted for 1 hour in order to provide enough time for the dispersed JSL and the test product mixture to run out of the tube. Due to the high viscosity (impregnation effect) of some of the products, small amounts of the preparations were still attached to the vial after it was inverted. The weight of the tubes was determined (Test 1) again to calculate the exact amount of remaining JSL (ceruminolysis) and attached ear products (impregnation).

To gain comparable results to Sánchez-Leal et al. [17] and to simulate consecutive applications of the ear products, this procedure was repeated three times (Tests 2–4). The weight of the tubes before each test run was set as 100%. Due to the high viscosity of some of the products, water was used to remove all test preparations left in the tubes in the final test.

In the present study, the ceruminolytic activity was defined as the weight loss of JSL after consecutive incubation and rinsing of otic preparations (Tests 1–4). Ceruminolysis causes disintegration and elimination of cerumen and so will be characterised by weight loss. Conversely, any increase in weight would indicate an impregnation of the product in the JSL. Four loadings of each otic preparation were run simultaneously. Water was used as a negative control with low variability.

### Statistics

The differences in the quantities of the lipids between OE cerumen and cerumen of healthy dogs were statistically analysed by *t*-test followed by the Mann Whitney test as post-hoc test. A level of significance of p < 0.05 was adopted.

The differences in the diffusion rates after 24 hours and the percentages of ceruminolytic removal were statistically analysed by One-Way Analysis of Variance (ANOVA) with Tukey’s multiple comparison test as post-hoc test. A level of significance of p < 0.05 was adopted. The software GraphPad Vision 5.0 (Graphpad Software, Inc., La Jolla, USA) was used for the analyses.

## Abbreviations

HPLC: High performance liquid chromatography; JSL: Synthetic cerumen; OE: Otitis externa; SCC: Synthetic canine cerumen; TLC: Thin layer chromatography.

## Competing interests

WRP is an employee of Vétoquinol GmbH, Germany.

## Authors’ contributions

JS conducted the diffusion experiments using the canine cerumen described in the literature and the ceruminolytic study, contributed to the analysis, interpreted the results and drafted the manuscript. SM conducted the thin layer chromatography and the diffusion experiments with SCC. WRP helped to draft the manuscript. MK participated in the study design and coordination and helped to draft the manuscript. All four authors read and approved the final manuscript.
